# Training-Induced Muscle Fatigue with a Powered Lower-Limb Exoskeleton: A Preliminary Study on Healthy Subjects

**DOI:** 10.3390/medsci10040055

**Published:** 2022-09-26

**Authors:** Renato Baptista, Francesco Salvaggio, Caterina Cavallo, Serena Pizzocaro, Svonko Galasso, Micaela Schmid, Alessandro Marco De Nunzio

**Affiliations:** 1 Department of Research and Development, LUNEX International University of Health, Exercise and Sports, Avenue du Parc des Sports, 50, 4671 Differdange, Luxembourg; 2 Luxembourg Health & Sport Sciences Research Institute ASBL, Avenue du Parc des Sports, 50, 4671 Differdange, Luxembourg; 3 Department of Public Health, Experimental and Forensic Medicine, University of Pavia, S. da Nuova, 65, 27100 Pavia, Italy; 4 Department of Electrical, Computer and Biomedical Engineering, University of Pavia, S. da Nuova, 65, 27100 Pavia, Italy; 5 Department of Electrical and Information Engineering, University of Cassino and Southern Lazio, via Gaetano Di Biasio, 43, 03043 Cassino, Italy

**Keywords:** rehabilitation, powered lower-limb exoskeleton training, force reduction, fatigue

## Abstract

Powered lower-limb exoskeletons represent a promising technology for helping the upright stance and gait of people with lower-body paralysis or severe paresis from spinal cord injury. The powered lower-limb exoskeleton assistance can reduce the development of lower-limb muscular fatigue as a risk factor for spasticity. Therefore, measuring powered lower-limb exoskeleton training-induced fatigue is relevant to guiding and improving such technology’s development. In this preliminary study, thirty healthy subjects (age 23.2 ± 2.7 years) performed three motor tasks: (i) walking overground (WO), (ii) treadmill walking (WT), (iii) standing and sitting (STS) in three separate exoskeleton-based training sessions of 60 min each. The changes in the production of lower-limb maximal voluntary isometric contraction (MVIC) were assessed for knee and ankle dorsiflexion and extension before and after the three exoskeleton-based trained motor tasks. The MVIC forces decreased significantly after the three trained motor tasks except for the ankle dorsiflexion. However, no significant interaction was found between time (before-, and after-training) and the training sessions except for the knee flexion, where significant fatigue was induced by WO and WT trained motor tasks. The results of this study pose the basis to generate data useful for a better approach to the exoskeleton-based training. The STS task leads to a lower level of muscular fatigue, especially for the knee flexor muscles.

## 1. Introduction

Powered lower-limb exoskeletons are a new and emerging technology representing a promising solution for rehabilitating gait in various situations such as regaining locomotion ability and addressing gait impairments [1]. This technology has been studied and adopted in gait rehabilitation due to the increasing number of patients with gait impairments caused by spinal cord injury (SCI), stroke, Parkinson’s disease, neurological problems, or other related conditions [2,3]. Recovering locomotion is a main priority considering gait impairments [4,5]. Rehabilitation based on powered lower-limb exoskeletons allows for prolonged training sessions and personalised training by adapting the available movement patterns and adjusting the relative motion parameters [6,7].

People who experience gait impairment, either because of paresis or paralysis, often rely on using a wheelchair. At a global level, it is estimated that 65 million people would benefit from a wheelchair with an increasing prevalence of use partly due to the ageing of the population [8,9]. The wheelchair allows regaining the possibility to move, but long-time use progressively affects the musculoskeletal (i.e., decreasing body mineralization and quickening muscle loss [10]) and the cardiovascular [11] system leading even to psychological morbidity such as depressive symptoms [12]. Spasticity symptoms combined with the sedentary lifestyle of long-term wheelchair users remarkably decrease the quality of life (QoL) [13], with fatigue as a risk factor for developing such a vicious circle. This negative loop makes the onset of fatigue easier and quicker, even in performing ordinary activities of daily living (ADL) [14].

Fatigue brings a decrease in performance, but different causes lead to such an effect [15,16]. According to Barsotti et al. (2020) [17], muscular fatigue can be defined as the manifestation of a reduced maximal force or power during a muscle contraction. Keeping track of the fatigue level can prevent extenuating conditions induced by the training, allowing the body to recover between training sessions [17].

The main objective of this work is to assess lower-limb muscle fatigue induced by prolonged training based on a powered lower-limb exoskeleton during (i) walking overground (WO), (ii) treadmill walking (WT), (iii) standing and sitting (STS). Maximum voluntary isometric contraction (MVIC) has been used to assess the development of muscular fatigue across four different lower-limb muscle groups depending on the trained motor task (WO, WT, STS). As a preliminary study, healthy participants have been involved to provide a better understanding of the level of muscular fatigue induced by training with a powered lower-limb exoskeleton, depending on the specific executed motor task. The results of this study will provide initial information for future development of training protocols aiming at reducing the risk of spasticity by reducing the training-induced muscular fatigue.

## 2. Materials and Methods

### 2.1. Subjects

Thirty-four young and healthy subjects were screened to participate in this study. From the thirty-four, two were excluded due to the inclusion/exclusion criteria. Two other subjects were unreachable after the initial screening. At the end, a total of thirty young and healthy subjects were enrolled in this study. The subjects were contacted via email and provided with all the needed information about the study. After a first enrolment screening, based on the Health/Fitness Facility Pre-Participation Screening Questionnaire [18] and following the inclusion and exclusion criteria of the study (Table 1), the subjects were enrolled by signing the informed consent. After that, the subjects were asked to fill in the International Physical Activity Questionnaire (IPAQ) [19]. The IPAQ provides the level of physical activity which is expressed in a metabolic equivalent task in minutes per week (MET-minutes/week). IPAQ outcome indicates three categories of activity: inactive (<600 MET-minutes/week); minimally active (≥600 and<3000 MET-minutes/week); and health enhancing physical activity (HEPA active) (≥3000 MET-minutes/week) [19]. The baseline characteristics of the participants are presented in Table 2 and Table 3. A Mann-Whitney U test revealed no differences in gender distribution in terms of age and physical activity level. Height and weight were significantly different between the gender as expected [20]. A Chi-square test across the physical activity levels distribution resulted in a non-significant difference for a such parameter (*p* = 0.427, Table 3). The study was approved by the Comité National d’Ethique de Recherche (CNER-Luxembourg National Research Ethics Committee) with protocol number 202103/02, according to the Helsinki Declaration.

### 2.2. Study Design

**Figure 1:**
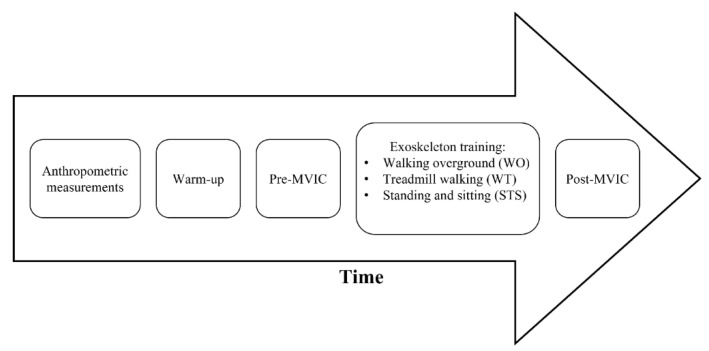
Study timeline description. MVIC—Maximal Voluntary Isometric Contraction.

A cross-sectional interventional study has been conducted to assess the changes in the lower limb muscle force production after receiving a lower-limb powered exoskeleton-based training. The training was based on executing three different motor tasks via the worn powered lower-limb exoskeleton (ExoAtlet, Esch-sur-Alzette, Luxembourg) during three separate training sessions: (i) walking overground (WO), (ii) treadmill walking (WT), (iii) standing and sitting (STS). Each participant performed 60 min exoskeleton-based training, across the three motor tasks, within at least an interval of 24 h between the training days. MVIC tests were performed before (pre) and right after (post) each exoskeleton-based training. Figure 1 shows an overview of the proposed study design.

For convenience and consistency, the participants were asked to wear the same sports clothes and shoes during the three training sessions. At the first training session, (i) height, (ii) weight, (iii) hip-to-hip distance, (iv) greater trochanter to the lateral epicondyle (bilaterally), (v) lateral epicondyle to foot sole were recorded and used to adjust the exoskeleton to the participant’s body measures characteristics.

**Figure 2:**
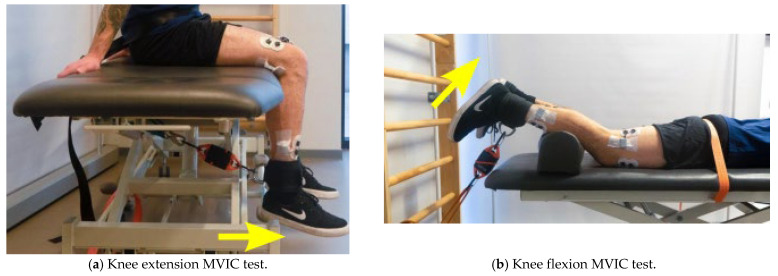

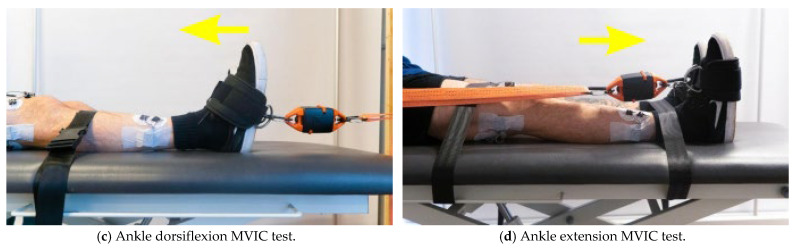
Pre and Post MVIC tests for the tested joints. The yellow arrow represents the direction of the movement to be performed. MVIC—Maximal Voluntary Isometric Contraction.

Before starting each training session, the participants warmed-up with a 10 min treadmill walk set at a fast-walking pace (from 4 to 6 km/h). After the warm-up, the pre MVIC test was performed for the knee and ankle dorsiflexion and extension of the dominant leg [21]. MVIC testing setup is reported in Figure 2. An electronic dynamometer (K-Link, K-Invent, Montpellier, France) was used to acquire the MVIC forces. The order of the tested body segments’ motion was randomized for the pre MVIC test using a free randomization software app (Random Generator, Apps n Blue, Jordan). The MVIC test consisted of 3 contractions of 5-s with a 60-s break for each contraction [22]. The participants were asked to maintain the maximum intensity for the whole duration of the contraction (5-s).

Right after the pre MVIC test, the experimenters helped the participants in donning the powered lower-limb exoskeleton and started the 60 min training session with a 5 min break at mid-time. The order of the trained motor tasks was randomized. WO task consisted of walking straight, at an average speed of 1.3 km/h, along a 10 m by 1 m path created using two separate rehabilitation parallel bars for safety. Two rotational platforms for patient mobilization (Disco Duo, Chinesport SPA, Udine, Italy) were placed at the extremities of the walking path to facilitate and speed the rotation of the powered lower-limb exoskeleton up during walking to and from. WT task consisted of walking on a rehabilitation treadmill (Gait Trainer 3, BIODEX, Shirley, NY, USA) with the same average constant speed used during the WO training (1.3 km/h). The treadmill was fitted with safety lateral bars. STS task consisted of continuously sitting and standing, starting from a standing position, for the 60 min duration of the training. Two lateral bars were always present for safety reasons. The experimenters executed each donning and doffing of the powered lower-limb exoskeleton with the participant sitting (i) in front of the walking path for WO, (ii) on the treadmill for the WT and (iii) on a chair adjusted in height to allow a 90 degrees knee flexion while sitting for the STS. Two experimenters constantly supervised the participants while standing or walking close to their side during the exoskeleton-based training. A third experimenter held the “control handles” on the back of the exoskeleton. The control handles allowed the experimenter to start and stop the motor tasks when needed, e.g., while turning during WO, for the mid-term break, to swap from sitting to standing during the STS. No consistent support from the experimenter controlling the powered lower-limb exoskeleton was needed unless a participant unbalance was present (which never happened during this study). The powered lower-limb exoskeleton provided all the needed support and motion to the body of the wearing participants who used the lateral bars mainly for balance.

The post MVIC test was performed right after each exoskeleton-based training. The experimenters helped the participants to doff the powered lower-limb exoskeleton and start the MVIC test as fast as possible. The post MVIC consisted of 1 maximal contraction of 5 s per body segment with a 60 s break per contraction. The same pre MVIC order test sequence was kept.

#### ExoAtlet II

**Figure 3:**
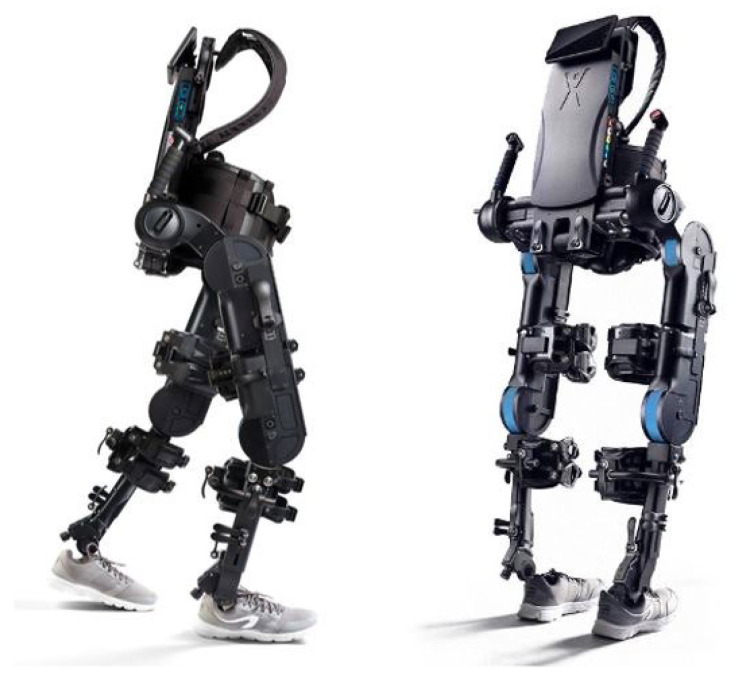
Powered lower-limb exoskeleton ExoAtlet II from ExoAtlet Global SA, Luxembourg. Adapted with permission from [23]. Copyrighted by ExoAtlet, 2019.

The powered lower-limb exoskeleton model used in this study was the ExoAtlet II from the ExoAtlet Global SA, Luxembourg [23] (see Figure 3). ExoAtlet II application field is healthcare as it is designed to assist lower limb motor functions of people with walking disabilities of neurological or musculoskeletal nature. ExoAtlet II includes a metallic exoskeleton structure, electric motors, mechanical actuators, an onboard computer and smart crutches for control and safety. Four motors drive the hip and the knee bilaterally, providing 2 degrees of freedom each. ExoAtlet II ankle joint operates in a passive mode.

The powered lower-limb exoskeleton is adjustable for people within 160–190 cm height and up to 100 kg of weight, strapped to the user with thoracic and lumbar belts, femoral and tibial straps, and a protecting sciatic belt. Powered lower-limb exoskeleton adjustments include shank and thigh length, pelvis width and depth. Adjustable walking parameters include step length, speed and height, and the time delay between steps. Available operating modes are standing, stepping in place, walking, standing up and sitting down on a chair. Batteries last for four continuous hours in walking operating mode. The powered lower-limb exoskeleton measures vary from 105 to 150 cm in height, 43 to 60 cm in width and 27 to 30 cm in depth. ExoAtlet II weights up to 32 kg. ExoAtlet II technology readiness level is 9 (full commercial application). ExoAtlet II description, set-up and applications have been further explored by Pais-Vieira et al. (2020) [24]. Table 4 shows the exoskeleton kinematics parameters set in this study as the device pre-set parameters for the tested subjects. The indicated set of parameters was chosen to keep a natural gait pattern for all the participants following the methods in [24].

### 2.3. Statistical Analysis

A within-subject design, with two factors comparison: (i) time pre and post MVIC tests, and (ii) trained motor tasks, WO, WT, and STS was used. All the acquired data are presented as means and standard deviations. A Two-Way Repeated Measures Analysis of Variance (RM-ANOVA) was used for normally distributed data (checked via the Shapiro-Wilk test). A corresponding non-parametric test (Friedman) was used in case of non-normal data distribution. A Bonferroni, or a non-parametric Conover, post hoc test was performed when the RM-ANOVA, or Friedman, showed statistically significance. The level of statistically significance was set at α=0.05. All the statistics were performed using the IBM SPSS Statistics software, version 28.

## 3. Results

**Figure 4:**
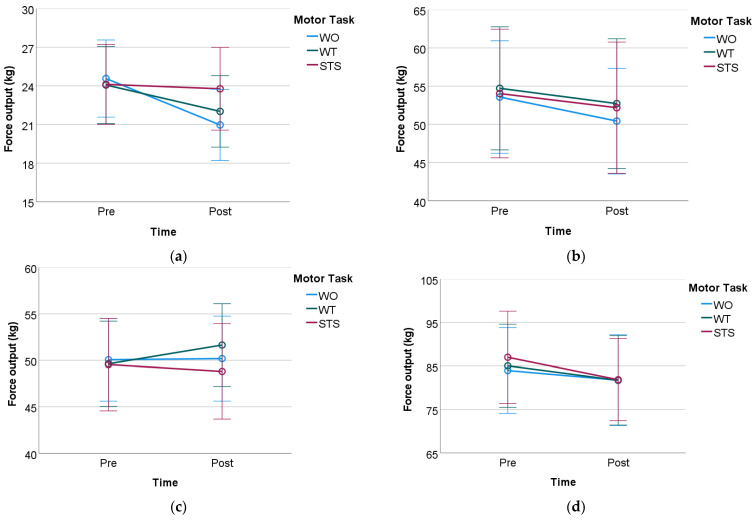
Mean and standard deviation of the pre and post MVIC test for all the three motor tasks. WO—Walking overground; WT—Treadmill walking; STS—standing and sitting. (**a**) Knee flexion; (**b**) Knee extension; (**c**) Ankle dorsiflexion; (**d**) Ankle extension.

Table 5 reports the descriptive statistics for all the motor tasks and time condition (pre and post) of the MVIC tests performed during the study. The results and corresponding RM-ANOVA tables are presented below for each MVIC body segment test. Figure 4 shows the variation of mean force output of the pre and post MVIC per each motor task.

### 3.1. Knee Flexion MVIC Test

Table 6 presents the main effects for the time factor (pre vs. post training), $F_{1,29}=17.68,\;p<0.001$, and no significant difference across the three motor tasks, $F_{2,29}=1.78,\;p=0.178$. A statistically significant interaction was observed between time and motor task, $F_{2,29}=5.937,\;p=0.005,\;\eta_{p}^{2}=0.17$. The Bonferroni post hoc test was performed (Table 7) showing a statistically significant result for the interaction for WO and WT, p=<0.001, 95% C.I. =[2.072, 5.135], and p=0.008, 95% C.I. =[0.568, 3.526], respectively. As for the STS motor function task, no statistically significant difference was observed in the interaction with the time factor, p=0.625, 95% C.I.=[−1.084, 1.775].

### 3.2. Knee Extension MVIC Test

A statistically significant effect on the force output on the time factor, $F_{1,29}=10.461,\;p=0.003,\;\eta_{p}^{2}=0.265$ (Table 8). In contrast, no significant effect was observed for the three motor function tasks or the interaction between the two factors.

### 3.3. Ankle Dorsiflexion MVIC Test

No statistically significant difference was observed for the time, $F_{1,29}=0.349,\;p=0.559$, or for the three motor tasks factor, $F_{2,29}=0.629,\;p=0.537$. No statistically significant interaction between time and motor tasks was found, $F_{2,29}=1.825,\;p=0.17$, see Table 9.

### 3.4. Ankle Extension MVIC Test

RM-ANOVA shows a statistically significant effect on the force output on the time factor, $F_{1,29}=8.817,\;p=0.006,\;\eta_{p}^{2}=0.233$. No statistically significant effects of the force output were observed across the three motor tasks nor the two factors interaction, see Table 10.

## 4. Discussion

Generally speaking, the walking or standing and sitting powered lower-limb exoskeleton training induces muscular fatigue at the knee and ankle level, except for the ankle dorsiflexor muscles. The study results showed a significant general drop in the MVIC output force induced by the exoskeleton training. However, such force reduction was not relevant for the ankle dorsiflexion MVIC test as the exoskeleton-based training across the three trained motor tasks led to no muscle fatigue at the ankle level when dorsiflexing (e.g., tibialis anterior). The exoskeleton-based training was, therefore, inducing significant muscle fatigue across those body segments insisting on an ankle extension (e.g., calf muscles), knee flexion (e.g., the hamstrings) and knee extension (e.g., quadriceps). There was no clear indication on which of the trained motor tasks (WO, WT, STS) induced a more consistent level of fatigue in the three latter sets of body segments apart for those insisting on the knee flexion. This study indicates that long-lasting exoskeleton training (similarly to the study presented in [25]), on walking overground or on a treadmill, induces fatigue in the knee flexor muscles (e.g., the biceps femoris).

A possible reason for that comes from the mechanical design of the exoskeleton. The exoskeleton is a series of rigid frames imposing a continuously repeated precise gait pattern which actively moves the knee and hip in the sagittal plane. The hip motions around the transversal and frontal plane are somehow limited even if important during gait [26]. We suppose that the obtained higher level of fatigue for the hamstring muscles can originate from the constraints imposed by the exoskeleton at the hip level [27]. All the baseline characteristics of the study group were statistically similar apart from the height and weight of the subjects. No differences in fatigue level are expected by different levels of physical activity across participants. The fatigue induced by the exoskeleton training cannot be ascribed to the differences in height and weight as the mechanical support and the power outcome provided by the exoskeleton is the same for any user weighting lower than 95 kg and a height lower than 190 cm.

This study provides preliminary evidence-based indications about the level of fatigue at the lower limb induced while using a powered lower-limb exoskeleton. The results obtained should be taken cautiously before considering moving toward clinical practice. The limitations of this study come from the non-clinical population investigated and the induced fatigue measured only via force data. A future series of studies will consider a clinical neurological population (e.g., SCI, and multiple sclerosis) and enrich the fatigue assessment via superficial muscular activity (sEMG) to deepen the understanding of the level of muscular fatigue developed during the trained motor tasks.

Considering the above reported limitations, according to the study results, particular attention should be used while training the gait of patients subjected to developing spasticity at the lower limb muscles using a powered lower-limb exoskeleton. Such training can lead to significant fatigue and spasticity in the knee flexor muscles. On the other hand, the ankle dorsiflexor muscles are the least susceptible to fatigue during the exoskeleton-based training of gait, standing and sitting functions. Based on the study results, a general indication of the motor tasks to train using a powered lower-limb exoskeleton can be drafted. Standing and sitting exoskeleton-based training can be preferred as less fatiguing for the knee flexors than exoskeleton-based gait training. The walking overground and treadmill walking tasks induce a consistent reduction in the MVIC output force for knee flexion, as a consequence of hamstring muscle induced fatigue. Such a result provides evidence for better use of an active powered lower-limb exoskeleton especially if combined with additional rehabilitation exercises. The obtained results indicate avoiding or constantly checking for fatigue development when administering the exoskeleton-based walking training jointly with knee flexors exercises [28,29,30].

### Statistical Considerations for Further Studies

The effect size of the non-statistically significant results varies between small (usually around $\eta_{p}^{2}=0.01$), small to medium, and medium (usually around $\eta_{p}^{2}=0.06$) at best. On the other hand, the statistically significant results are supported by medium to large or large (usually $\eta_{p}^{2}>0.14$) effect size. Such observations suggest that increasing the study’s sample size would change the non-significant results.

## 5. Conclusions

The study assessed the lower limb muscle fatigue induced by a long-last powered lower-limb exoskeleton-based training across three motor tasks (namely walking overground, treadmill walking, standing and sitting from a chair). Fatigue was assessed via MVIC tests before and after the exoskeleton-based training for the knee, and ankle dorsiflexion, and extension. The study concludes that both walking overground and on a treadmill with a powered lower-limb exoskeleton induce muscle fatigue in the knee flexors (e.g., hamstrings).

The study results indicate avoiding exercising the hamstring muscles in the same gait training session involving a powered lower-limb exoskeleton to build up excessive fatigue in such muscles. Further studies involving a larger sample size and an extension of the sample size to subjects with neurological impairments are advised. Such next steps will seek indications towards the optimal intensity that allows a powered lower-limb exoskeleton-based rehabilitation to produce positive effects avoiding building up muscle fatigue in the lower limbs and increasing the risk for spasticity. Future studies will promote the development of standardized and evidence-based training protocols based on powered lower-limb exoskeletons.

## Figures and Tables

**Table 1:** Inclusion and exclusion criteria.

Inclusion Criteria	Exclusion Criteria
	Currently seeking treatment;
Age between 18–35, as broadly defined as adulthood;	Currently pregnant;
Weight < 95 kg (as an imposed limit for the exoskeleton training);	Individuals affected by systemic, rheumatic, or neuro-musculoskeletal disorders;
Height between 150 and 195 cm (as an imposed limit for the exoskeleton training);	Current use of anti-anxiety medications, muscle relaxants or tranquillizers;
Ability to exercise safely without medical approval (checked via the Health/Fitness Facility Pre-Participation Screening Questionnaire);	Relevant history of back or lower limb pain over the last three years;

**Table 2:** Participants’ baseline characteristics.

	Male	Female	Overall	*p*-Value (Mann-Whitney U Test)
Number	15	15	30	
Age (mean ± SD)	24 ± 3.2	22.3 ± 1.7	23.2 ± 2.7	0.164
Height (mean ± SD, cm)	180.7 ± 5	164.6 ± 5.5	172.7 ± 9.7	<0.001
Weight (mean ± SD, kg)	78.7 ± 9.8	58.3 ± 8.4	68.5 ± 13.7	<0.001
IPAQ (median (IQR), MET-minutes/week)	6960 (5152)	3342 (6604)	5055 (6880)	0.237

SD—standard deviation. IQR—interquartile range. MET-minutes/week—metabolic equivalent task in minutes per week.

**Table 3:** Participants’ baseline characteristics in terms of physical activity levels (IPAQ categories).

	IPAQ Category			
Gender	Inactive	Minimally Active	HEPA Active	Total
Male	0	3	12	15
Female	0	6	9	15
Total	0	9	21	30

**Table 4:** Exoskeleton parameters for the motor function tasks.

Task	Setup Parameters	Value
Walking overground	Pause between steps (s)	0
&	Step length (cm)	40
Treadmill walking	Step height (cm)	15
	Step duration (s)	1.2
Stand-up sit-down	Standing up duration (s)	3
	Sitting down duration (s)	2

**Table 5:** Descriptive statistics of the pre and post MVIC tests per muscle and motor task.

Motor Task	Time	Knee Flexion ± SD (kg)	Knee Extension ± SD (kg)	Ankle Dorsi Flexion ± SD (kg)	Ankle Extension ± SD (kg)
WO	Pre	24.57 ± 8.01	53.58 ± 19.71	50.06 ± 11.94	83.96 ± 26.4
	Post	20.97 ± 7.41	50.43 ± 18.51	50.18 ± 12.25	81.76 ± 27.95
WT	Pre	24.07 ± 7.98	54.72 ± 21.56	49.63 ± 12.29	85.05 ± 25.6
	Post	22.02 ± 7.44	52.72 ± 22.73	51.64 ± 11.93	81.71 ± 27.56
STS	Pre	24.12 ± 8.31	54.03 ± 22.52	49.55 ± 13.33	87.01 ± 28.43
	Post	23.77 ± 8.6	52.18 ± 22.99	48.81 ± 13.74	81.87 ± 25.3

WO—Walking overground; WT—Treadmill walking; STS—standing and sitting; SD—standard deviation.

**Table 6:** Two-way ANOVA—Knee flexion MVIC test.

Repeated Measures Two-Way ANOVA—Within-Subjects Effects					
Cases	df	Mean Square	F	p	$\eta_{p}^{2}$
Motor task	2	22.8	1.78	0.178	0.058
Time	1	179.74	17.68	<0.001 ^†^	0.379
Motor task x Time	2	39.83	5.937	0.005 ^†^	0.17

^†^ Significant difference at p<0.05.

**Table 7:** Bonferroni post hoc test.

Pairwise Comparisons Motor Task × Time							
Motor Task	Time (I)	Time (J)	Mean Diff. (I-J)	Std. Error	p	95% Confidence Interval for Difference ^a^	
						Lower Bound	Upper Bound
WO	Pre	Post	3.603	0.749	<0.001 ^†^	2.072	5.135
WT	Pre	Post	2.047	0.723	0.008 ^†^	0.568	3.526
STS	Pre	Post	0.345	0.699	0.625	−1.084	1.775

WO—Walking overground; WT—Treadmill walking; STS—standing and sitting. ^a^ Adjustment for multiple comparisons: Bonferroni ^†^ Significant difference at p<0.05.

**Table 8:** Two-way ANOVA—Knee extension MVIC test (vastus lateralis).

Repeated Measures Two-Way ANOVA—Within-Subjects Effects					
Cases	df	Mean Square	F	p	$\eta_{p}^{2}$
Motor task	2	45.25	0.482	0.62	0.016
Time	1	244.43	10.461	0.003 ^†^	0.265
Motor task x Time	2	7.528	0.38	0.686	0.013

† Significant difference at *p* < 0.05.

**Table 9:** Two-way ANOVA—Ankle dorsiflexion MVIC test.

Repeated Measures Two-Way ANOVA—Within-Subjects Effects					
Cases	df	Mean Square	F	p	$\eta_{p}^{2}$
Motor task	2	32.83	0.629	0.537	0.021
Time	1	9.73	0.349	0.559	0.012
Motor task x Time	2	29.72	1.825	0.17	0.059

**Table 10:** Two-way ANOVA—Ankle extension MVIC test.

Repeated Measures Two-Way ANOVA—Within-Subjects Effects					
Cases	df	Mean Square	F	p	$\eta_{p}^{2}$
Motor task	2	38.75	0.339	0.714	0.012
Time	1	569.23	8.817	0.006 ^†^	0.233
Motor task x Time	2	32.99	0.853	0.431	0.029

^†^ Significant difference at p<0.05.

## Data Availability

The data presented in this study may be available upon request from the corresponding author.
